# Proteome Profiling of the Exhaled Breath Condensate after Long-Term Spaceflights

**DOI:** 10.3390/ijms20184518

**Published:** 2019-09-12

**Authors:** Alexey S. Kononikhin, Alexander G. Brzhozovskiy, Anna M. Ryabokon, Kristina Fedorchenko, Natalia V. Zhakharova, Alexander I. Spasskii, Igor A. Popov, Vyacheslav K. Ilyin, Zoya O. Solovyova, Lyudmila Kh. Pastushkova, Alexey V. Polyakov, Sergey D. Varfolomeev, Irina M. Larina, Evgeny N. Nikolaev

**Affiliations:** 1 Laboratory of mass spectrometry, CDISE, Skolkovo Institute of Science and Technology, 121205 Moscow, Russia; 2 Russian Federation State Scientific Research Center Institute of Biomedical Problems, Russian Academy of Sciences, 119991 Moscow, Russia; 3 Emanuel Institute for Biochemical Physics, Russian Academy of Sciences, 119991 Moscow, Russia; 4 Department of Chemistry, Lomonosov Moscow State University, 119991 Moscow, Russia; 5 Laboratory of ion and molecular physics, Moscow Institute of Physics and Technology, Dolgoprudny, 141701 Moscow, Russia; 6 V.L. Talrose Institute for Energy Problems of Chemical Physics, N.N. Semenov Federal Center of Chemical Physics, Russian Academy of Sciences, 119334 Moscow, Russia

**Keywords:** spaceflight, exhaled breath condensate, proteomics, mass-spectrometry, astronauts, extreme conditions

## Abstract

Comprehensive studies of the effects of prolonged exposure to space conditions and the overload experienced during landing on physiological and biochemical changes in the human body are extremely important in the context of planning long-distance space flights, which can be associated with constant overloads and various risk factors for significant physiological changes. Exhaled breath condensate (EBC) can be considered as a valuable subject for monitoring physiological changes and is more suitable for long-term storage than traditional monitoring subjects such as blood and urine. Herein, the EBC proteome changes due to the effects of spaceflight factors are analyzed. Thirteen EBC samples were collected from five Russian cosmonauts (i) one month before flight (background), (ii) immediately upon landing modules in the field (R0) after 169–199 days spaceflights, and (iii) on the seventh day after landing (R+7). Semi-quantitative label-free EBC proteomic analysis resulted in 164 proteins, the highest number of which was detected in EBC after landing (R0). Pathways enrichment analysis using the GO database reveals a large group of proteins which take part in keratinization processes (*CASP14*, *DSG1*, *DSP*, *JUP*, and so on). Nine proteins (including *KRT2*, *KRT9*, *KRT1*, *KRT10*, *KRT14*, *DCD*, *KRT6C*, *KRT6A*, and *KRT5*) were detected in all three groups. A two-sample Welch’s *t*-test identified a significant change in KRT2 and KRT9 levels after landing. Enrichment analysis using the KEGG database revealed the significant participation of detected proteins in pathogenic *E. coli* infection (*ACTG1*, *TUBA1C*, *TUBA4A*, *TUBB*, *TUBB8*, and *YWHAZ*), which may indicate microbiota changes associated with being in space. This assumption is confirmed by microbial composition analysis. In general, the results suggest that EBC can be used for noninvasive monitoring of health status and respiratory tract pathologies during spaceflights, and that the obtained data are important for the development of medicine for use in extreme situations. Data are available from ProteomeXchange using the identifier PXD014191.

## 1. Introduction

Space flight is one of the most extreme conditions encountered by humans. The combined effect of factors such as radiation, microgravity, hypodynamia, and isolation disturb homeostatic systems and affect a majority of physiological systems [1,2]. The factors associated with hypogravity during a prolonged space flight leads to the restructuring of all body systems, towards a new level of functioning, which includes a decrease in bone and muscle tissue, the redistribution of bodily fluids, and a number of other physiological changes that cannot be accompanied by changes in the regulation of vital activity, including gene expression and protein synthesis [3]. The monitoring of changes in individual physiological and biochemical characteristics is of particular importance for the development of personalized countermeasures, in order to minimize the risks associated with being in space, and many experimental tools and methods have been developed to study space-induced physiological changes [4]. The increased background radiation was shown by NASA and Roscosmos researchers to be one of the primary risk factors for the health of astronauts, which, together with constant overloads and age factors, increases the risk of developing cardiovascular diseases and oncology [5,6,7]. The Human proteomics at Extreme Conditions Initiative started in 2013 as an international collaborative initiative aiming to accumulate proteomic data on the effects of space flight factors on the human body, including both real space missions and ground-based model experiments (https://hupo.org/Human-Proteomics-at-Extreme-Conditions). However, to date, the study of proteomic space-flight associated changes have mostly been limited to the models of cultured cells, plants, Micro-organisms, and animals of various taxonomic groups [8,9,10,11,12]. Therefore, the study of spaceflight effects on changes in the human proteome remains very relevant.

Blood and urine are the primary traditional subjects for the screening of physiological changes associated both with various diseases, and with extreme conditions or environmental factors [13,14]. However, the proteome of these samples collected in space cannot be analyzed in short time, and long-term storage significantly reduces their nativeness. Exhaled breath condensate (EBC) is another biological fluid which can be collected noninvasively, as frequently as necessary, and can be stored at −80 °C for up to 8 months [15]. As respiratory function is vital, and both lifestyle and health status significantly affect the state of the respiratory system, EBC can be an essentially important subject for monitoring physiological changes, providing valuable information for rapid diagnostics of the state of the respiratory system state [16,17,18,19]. It typically includes up to 2000 compounds of various classes, including proteins, lipids, antibodies, carbohydrates, amino acids, and other non-volatile biomacromolecules [17,20,21]. Currently, nitrous oxide (NO) [22,23,24,25], hydrogen peroxide (H_2_O_2_) [26,27,28,29], and acetone are conventional and FDA-approved markers for the diagnosis of inflammatory processes in the respiratory system. Lipid components, steroids, eicosanoids, and their subclasses of prostaglandins and isoprostanes have been shown to be possible markers of various pathologies [30,31]. In particular, 8-isoprostane is considered to be a biomarker of oxidative stress [32] and the pH of EBC can be considered as a simple but reliable biomarker of various lung diseases [33,34,35]. A number of proteins including cytokeratins (1, 5, 9, and 14), C-reactive protein, interleukins (IL-1α, IL-1β, IL-2, IL-12α, IL-12β, and IL-15), tumor necrosis factor-α, C3 complement component, and interferons α and γ, as well as proteome profiles have been shown to be characteristic for particular pathologies, including asthma, chronic obstructive pulmonary disease, lung cancer, and so on [36,37,38]. However, most useful for the present study is the fact that EBC composition reflects the real-time state of the respiratory system. As the conditions of space flight and landing create an extraordinary load, the systemic changes in the human organism can also affect the composition of the EBC, including its proteome [4].

In this study, a comparative semi- quantitative label-free EBC proteome analysis of samples collected from cosmonauts before and after long-term space flights was performed. The results suggest that EBC analysis may provide important information on systemic changes in the human organism, and can be used for noninvasive monitoring of the health status and respiratory tract pathologies of astronauts during the spaceflight.

## 2. Results and Discussion

**Figure 1:**
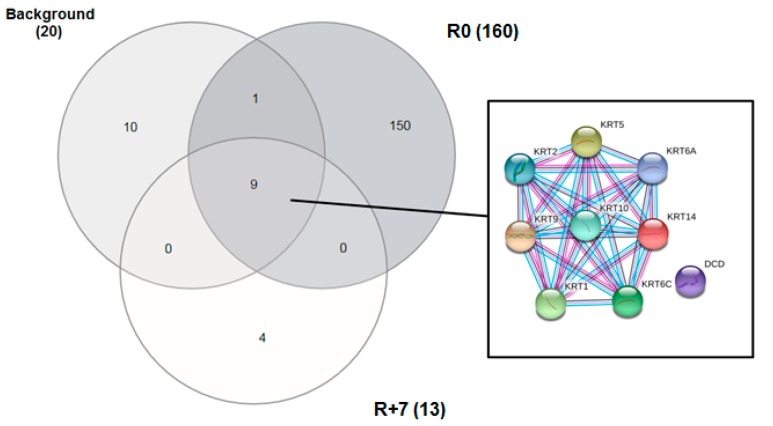
Venn diagram of 164 proteins identified in exhaled breath condensate (EBC) samples in three different groups: Background (1 month before landing), R0 (immediately after landing) and R+7 (seventh day after landing). For the 9 proteins detected in all groups, a STRING proteins interaction network is presented: Purple indicates experimentally determined interactions, blue indicates interactions from the curated database and black indicates co-expression of genes.

A semi-quantitative label-free proteomic analysis of 13 EBC samples collected from five Russian cosmonauts before and after long-term (169–199 days) spaceflights was performed. EBC samples were collected at the Yu. A. Gagarin Research and Test Cosmonaut Training Center one month before flight (background), immediately after landing (R0), and on the seventh day after landing (R+7). LC-MS/MS analysis resulted in 164 different proteins. To analyze the correlation between samples and technical runs, Pearson’s coefficient was calculated. It showed good correlation (about 0.9) between sample runs and an acceptable correlation for inter-individual variability in the groups of samples. The distribution of the number of identified proteins between the background and R7 samples is presented in the Venn diagram (Figure 1).

Nine proteins (*KRT2*, *KRT9*, *KRT1*, *KRT10*, *KRT14*, *DCD*, *KRT6C*, *KRT6A*, and *KRT5*) were detected in all groups (Table 1). Interaction analysis using the STRING database [39] showed that eight of the proteins belong to intermediate and keratin filaments. *KRT14*, *KRT2*, *KRT5*, *KRT6A*, and *KRT9* function as structural constituents of the cytoskeleton and *KRT1*, *KRT10*, and *KRT2* are structural constituents of epidermis. Analysis of proteome composition shows that the highest number of proteins was detected in the R0 group (Figure 1, Supplementary table).

Enrichment analysis using the KEGG database revealed significant participation of detected proteins (*ACTG1*, *TUBA1C*, *TUBA4A*, *TUBB*, *TUBB8*, and *YWHAZ*) in pathogenic *E. coli* infection. Additionaly, dermcidin was detected in the R0 group, which normally displays antimicrobial activity and is highly effective against *E.coli*, *E. faecalis*, *S. aureus*, and *C. albicans* [40]. As well as *KRT1* and *KRT6A*, *DCD* takes part in humoral immune response. Presumably, the presence of these proteins in EBC suggest changes in microbial composition. This assumption was confirmed by a microbial composition analysis. The microflora of the studied biotopes (mouth, dental plaque, tongue, and cheek) was represented by micro-organisms of the normal flora (Gram-positive micro-organisms). The highest level of microbial contamination was revealed during the spaceflight. A high presence of *E. coli* was also observed in samples taken in the middle of a flight. In some cases, the presence of methicillin sensitive *S. aureus* was observed at R+7 (Table 2). Subsequently, during the rehabilitation period, the quantitative and species composition of microflora was normalized.

The experimental data correlated well with previous results. The cosmonauts’ microbiocenoses on the first day after landing have been characterized by changes in the number of opportunistic pathogenic enterobacteria, clostridia, and lactobacilli (*p*-value > 0.01, *p*-value > 0.05, *p*-value > 0.05, respectively) [41]. In addition, dysbacteriosis have been recorded in the crew of the “Salyut” and “Mir” space stations after long-term space flights [41]. Shifts in microbial composition have also been detected during ground-based experiments. During a dry immersion experiment, the increase of bacterial colony-forming units (CFU) of conditionally pathogenic strains (*S. aureus* and *enterobacterium spp.*, *bacillus spp.*) and decrease of symbiotic microflora (*neiseria spp.* and *corinobacterioum spp.*) were detected [42]. 

Pathways enrichment analysis (using the GO processes and pathways database) showed that the majority of R0 proteins participate in innate immune system processes and neutrophil degranulation. The immunochemical analysis of gingival fluid in the examined test subjects showed a decrease in the level of immunoglobulins (sIgA, IgA, and IgM). A decrease in the sIgA level may indicate the possibility of an inflammatory process in the periodontal tissues [43].

**Figure 2:**
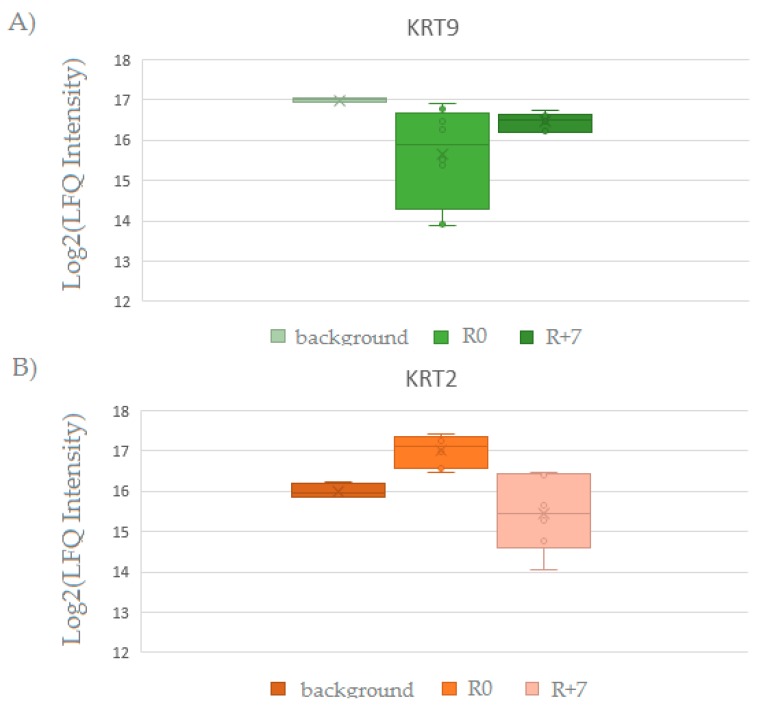
The label-free quantification (LFQ) intensity box plot for *KRT9* (**A**) and *KRT2* (**B**) proteins which changed their levels after landing, with respect to the background. The LFQ values are plotted on a Log2(x) scale along the vertical axis.

Some proteins also take part in keratinization (*CASP14*, *DSG1*, *DSP*, *JUP*, *KRT6B*, *PKP1*, *SFN*, *TGM1*, and *TGM3*). In previous studies cytoskeleton keratins were shown as to be main proteins in the EBC of healthy peoples [44,45]. Some of them are also associated with different pathologies of the respiratory system such as pneumonia and lung cancer [37,38,46]. Due to Protein Atlas, keratins *KRT1*, *KRT 2*, *KRT 9*, and *KRT 10* have epidermal origin and can contaminate samples during sample preparation. In previous studies, we have suggested keratins of EBC to belong to proteins circulating in the ambient air [19,44]. Almost every protein detected in EBC samples during background and in R+7 groups is typical for EBC, despite the conditions of the lung system. The keratins detected in each group are presented in Table 1. A two-sample Welch’s *t*-test was applied to identify the significantly changed proteins between the study groups. KRT2 increased and KRT9 decreased their respective levels after landing (Figure 2). In addition to their functions keratins are involve in cell signaling, communicating with extracellular matrix (ECM) components through desmosomes and hemidesmosomes [39].

Previously we showed that overexpression of stress- or stimulus-associated proteins can be detected in urine on the 1st day after landing [13]. Most of the urine significant proteins that are associated with stress factors returns to its preflight levels up to the 7th day. On other hand, EBC and blood plasma proteome analysis revealed significantly changing proteins associated with the immune response. It is worth noting that plasma proteome changes due to the space flight correlates with results of ground based experiments such as head-down bed rest (HDBR) and dry immersion experiments [47].

In general, the results of the study are in favor of further considering EBC as a subject for the non-invasive monitoring of systemic physiological changes during spaceflights, and analysis of a larger number of samples seems highly appropriate. Among other things, the obtained data may be important for the modernization of medical approaches in relation to extreme conditions.

## 3. Materials and Methods

### 3.1. Samples Collection

The study was performed within the “Protokon” experiment. It included five Russian cosmonauts of 38–62 years of age (the average age was 45.5 years; four males and one female). The biological samples were collected one month before long-term (169–199 day) spaceflight (background) at the Yu. A. Gagarin Research and Test Cosmonaut Training Center, immediately after landing of the landing modules in the field (R0), and on the seventh day after landing as a part of a medical examination (R+7). The EBC study was approved (Code «EBC proteome», date 06 March 2017) by the Ethics Committee of the Institute of Biomedical Problems, Russian Academy of Sciences/Physiology Section of the Russian Bioethics Committee Russian Federation National Commission for UNESCO and Human Research Multilateral Review Board, NASA, Houston, TX, USA. All donors signed the informed consent for participation in the study.

The EBC was collected using a portable RTube device [48]. During EBC collection, the cosmonaut breathed quietly, for 10–15 min, through a special mouthpiece with a salivary trap, and the exhaled airflow was diverted through a Teflon or polypropylene tube inside a cooling container. Immediately after collection samples were transported at −20 °C and stored at −80 °C until use. The sample preparation for LC-MS analysis included freeze-drying and selective tryptic hydrolysis at 37 °C for 17 h as previously discussed [49].

### 3.2. LC-MS/MS Proteomic Analysis

The tryptic peptide fraction (injection volume 1 µL) was analyzed in triplicate on a nano-HPLC Dionex Ultimate3000 system (Thermo Fisher Scientific, Waltham, MA, USA) coupled to a TiMS TOF mass spectrometry system (Bruker Daltonics, Bremen, Germany) using a captive spray ion source (positive ion mode, 1600 V) (Bruker Daltonics, Bremen, Germany). HPLC separation was performed on a C18 capillary column (25 cm × 75 µm × 1.6 µm) (Ion Optics, Parkville, Australia) at a flow rate of 0.4 µL/min by gradient elution. The mobile phase A was 0.1% formic acid in water and mobile phase B was 0.1% formic acid in acetonitrile. The separation was carried out by a 40 min gradient from 3–90% of phase B.

### 3.3. Data Analysis

MS data were analyzed using the MaxQuant (v 1.6.5) [50] program against the SwissProt Human database with an initial precursor mass error of 70 ppm. The minimum peptide length for identification was set to seven amino acids; the match between the runs option was activated. The cutoff false discovery rate (FDR) for proteins and peptides was set to 0.01 (1% FDR). Label-free quantitative analysis was performed in order to determine the significantly changed proteins. Quantification of peptides was recognized on the basis of mass and retention time but identified in other LC-MS/MS runs. Proteins quantification was carried out using the label-free quantification (LFQ) intensities of peptides across all samples and represented by a normalized intensity profile generated according to the specific algorithms. Protein–protein interactions were analyzed using the STRING database (v 11.0). The minimum coefficient of interaction score was 0.4; the PPI enrichment *p*-value was < 1.0 × 10^−16^. The interactions included physical and functional associations derived from computational prediction, automated text mining, co-expression databases and genomic context prediction aggregated from other databases [51]. Protein categorical annotations were derived from GeneOntology using the SwissProt Human database. The mass spectrometric proteomic data were deposited into the ProteomeXchange Consortium through the PRIDE [49] partner repository.

### 3.4. Collection of Microbiological Samples

The biological samples for microbial analysis were collected 45 days and 1 day before spaceflight, in the middle of the spaceflight (second half) immediately after landing (R0) and 7 days after flight (R+7). Microbial samples collection was performed using sterile fluoroplastic tubes with tampons. The test tube contained a capillary with a preservative which moistened the swab and ensured the preservation of the initial microflora composition for up to 5 days. Bacteria were grown (incubated for 48 h at 37 °C) using the following culture media: blood agar (to determine the total microbial number); endo agar (to quantify the growth of Gram-negative rods); mannitol-salt agar (for quantitative assessment of staphylococcus growth); and saburo agar (to quantify the growth of yeast and yeast-like micro-organisms).

## Figures and Tables

**Table 1:** List of proteins belongs to keratins family presented in three study groups: background (month before flight), R0, and R+7 groups.

Protein IDs	Protein Names	Gene Names	Background	1 Day After Landing	7 Day After Landing	Peptides
P04264	Keratin, type II cytoskeletal 1	*KRT1*	17.6 ± 1.5	18.4 ± 0.3	17.8 ± 0.6	34
P13645	Keratin, type I cytoskeletal 10	*KRT10*	16.2 ± 1.4	18.2 ± 0.4	16.1 ± 1.3	30
P02533	Keratin, type I cytoskeletal 14	*KRT14*	15.4 ± 0.1	15.1 ± 1.4	14.5 ± 0.1	23
P35908	Keratin, type II cytoskeletal 2 epidermal	*KRT2*	16 ± 0.2	17 ± 0.4	15.4 ± 0.9	29
P13647	Keratin, type II cytoskeletal 5	*KRT5*	14.1 ± 0.2	13.8 ± 1.1	13.6 ± 0.3	24
P02538	Keratin, type II cytoskeletal 6	*KRT6*	14.1 ± 0.1	14.5 ± 1.5	13.1 ± 0	25
P35527	Keratin, type I cytoskeletal 9	*KRT9*	17 ± 0	15.6 ± 1.2	16.5 ± 0.2	16

**Table 2:** List of Micro-organisms of selected biotopes (mouth, dental plaque, tongue, and cheek) collected from cosmonauts. Colony-forming units (CFU) are presented.

Biotopes	Micro-Organisms	CFU
45 days before spaceflight		
Mouth	*Enterobacterium sp.*	10^7^
	*Enterococcus sp.*	10^4^
Dental plaque	*Streptococcus sp.*	10^2^
	*Enterobacterium sp.*	10^5^
Tongue	*Enterobacterium sp*	10^3^
	*Streptococcus sp.*	10^2^
	*Enterococcus sp.*	10^4^
Cheek	*Staphylococcus sp.*	10^4^–10^5^
1 day before spaceflight		
Dental plaque	*Staphylococcus sp.*	10^2^
Tongue	*Enterococcus sp.*	10^6^
Cheek	*Staphylococcus sp.*	10^4^
During the spaceflight		
Mouth	*Staphylococcus sp.*	10^2^
Dental plaque	*Staphylococcus sp.*	10^3^–10^7^
Tongue	*Staphylococcus sp.*	10^2^
	*E.coli*	10^7^
Cheek	*Staphylococcus sp.*	~10^1^
Immediately after landing (R0)		
Mouth	*Enterococcus sp.*	10^1^–10^6^
	*Staphylococcus sp.*	10^1^
Dental plaque	*Enterococcus sp.*	10^3^–10^6^
	*Staphylococcus sp.*	10^3^
Tongue	*Staphylococcus sp.*	10^3^
	*Enterococcus sp.*	10^3^
Cheek	*Staphylococcus sp.*	~10^3^
7 day after landing (R+7)		
Mouth	*Enterococcus sp.*	10^3^–10^5^
	*S. Aureus*	10^4^ Met S
Dental plaque	*Staphylococcus sp.*	10^1^
	*Enterococcus sp.*	10^5^
Tongue	*Enterococcus sp.*	10^5^–10^8^
	*S. Aureus*	10^7^ Met S.
Cheek	*Staphylococcus sp.*	10^1^–10^6^
	*S. Aureus*	10^4^ Met S

## Supplementary Materials

Supplementary materials can be found at https://www.mdpi.com/1422-0067/20/18/4518/s1. All LC-MS/MS and proteomic data are available via ProteomeXchange with identifier PXD014191.
